# The prevalence, genetic complexity and population-specific founder effects of human autosomal recessive disorders

**DOI:** 10.1038/s41525-021-00203-x

**Published:** 2021-06-02

**Authors:** Qingyang Xiao, Volker M. Lauschke

**Affiliations:** Department of Physiology and Pharmacology, Karolinska Institutet, Stockholm, Sweden

**Keywords:** Molecular medicine, Risk factors

## Abstract

Autosomal recessive (AR) disorders pose a significant burden for public health. However, despite their clinical importance, epidemiology and molecular genetics of many AR diseases remain poorly characterized. Here, we analyzed the genetic variability of 508 genes associated with AR disorders based on sequencing data from 141,456 individuals across seven ethnogeographic groups by integrating variants with documented pathogenicity from ClinVar, with stringent functionality predictions for variants with unknown pathogenicity. We first validated our model using 85 diseases for which population-specific prevalence data were available and found that our estimates strongly correlated with the respective clinically observed disease frequencies (*r* = 0.68; *p* < 0.0001). We found striking differences in population-specific disease prevalence with 101 AR diseases (27%) being limited to specific populations, while an additional 305 diseases (68%) differed more than tenfold across major ethnogeographic groups. Furthermore, by analyzing genetic AR disease complexity, we confirm founder effects for cystic fibrosis and Stargardt disease, and provide strong evidences for >25 additional population-specific founder mutations. The presented analyses reveal the molecular genetics of AR diseases with unprecedented resolution and provide insights into epidemiology, complexity, and population-specific founder effects. These data can serve as a powerful resource for clinical geneticists to inform population-adjusted genetic screening programs, particularly in otherwise understudied ethnogeographic groups.

## Introduction

Autosomal recessive (AR) diseases constitute a subset of genetic disorders that are responsible for a considerable disease burden, affecting ~1.7–5 in 1000 neonates (compared to 1.4 in 1000 for autosomal dominant disorders)^1^. AR disease prevalence can be even substantially higher in populations with high rates of consanguinity or founder effects in combination with endogamy^2–4^. Thus, understanding the genetic underpinnings of AR diseases is of central importance for clinical genetics and public health. Firstly, information about major risk variants in a population facilitates newborn screening efforts to allow for early diagnosis of treatable disorders in the presymptomatic period, thereby allowing timely interventions to reduce patient morbidity and improve long-term health outcomes^5^. Secondly, carrier screening can identify couples at risk and provide a basis for genetic and reproductive counseling, which can drastically decrease disease incidence^6^.

By 2020, >2000 genetic disorders with AR inheritance have been described. However, for the vast majority of these diseases underlying genetic risk factors have not been systematically studied, and the available literature is limited to case reports. Furthermore, population-specific prevalence data for most diseases is lacking, which is particularly important as carrier frequency and incidence can drastically differ between ethnogeographic groups. For instance, Tay–Sachs disease (Online Mendelian Inheritance in Man (OMIM) identifier 272800), a lysosomal storage disorder caused by pathogenic variations in the *HEXA* gene is ~100 times more frequent in Ashkenazi Jews (1 in 3600 live births) compared to other populations (1 in 320,000). Similarly, highly population-specific variability in disease prevalence has been described for sickle cell anemia (OMIM 603903) and hemochromatosis (OMIM 235200).

AR diseases differ not only in frequency across populations, but also in their molecular genetics. For instance, the majority of cystic fibrosis (OMIM 219700) cases in Caucasians (72%) are explained by *CFTR* p.Phe508del, whereas the presumptive founder mutation p.Trp1282Ter was the most prevalent variant in Ashkenazim explaining 46% of cystic fibrosis cases (compared to 1.5% in Europeans)^7^. Furthermore, CFTR harbors >2000 additional variants, many of which also differ multiple fold in frequency across populations^8^. As a consequence of this genetic complexity, standardized genetic screening panels that do not consider interethnic differences in disease genetics can perform poorly in populations with specific founder effects^9^. While detrimental effects on test performance are already evident for well-studied diseases, such as cystic fibrosis, these problems are further exacerbated for diseases for which less is known about their underlying genetic variability.

Here, we conducted the most extensive analysis of the genetic variability underlying AR human diseases reported to date, covering 508 genes associated with 450 disorders based on next-generation sequencing data from 141,456 individuals across seven ethnogeographic groups. By integrating all pathogenic variants from ClinVar with highly stringent functionality predictions for rare and novel variants with unknown or conflicting pathogenicity annotations, we identified a total of 46,935 putatively disease-causing variations. Based on their variant frequencies, we modeled the incidences of all human AR diseases. We validated the method using 85 diseases with available prevalence data and showed that our model yielded accurate predictions of population-specific disease frequencies for monogenic disorders (*r* = 0.68; *p* < 0.0001). Using this resource for rare disease epidemiology, we provide quantitative ethnogeographic maps of human AR disease prevalence and pinpoint variant panels, with maximal test effectiveness to guide the design of population-specific carrier and newborn screening panels across seven populations. Furthermore, we calculated the genetic complexity of all 450 analyzed AR diseases and find >25 diseases with pronounced population-specific founder effects, most of which have not been previously described.

## Results

### The landscape of pathogenic variation associated with human autosomal recessive disorders

**Fig. 1:**
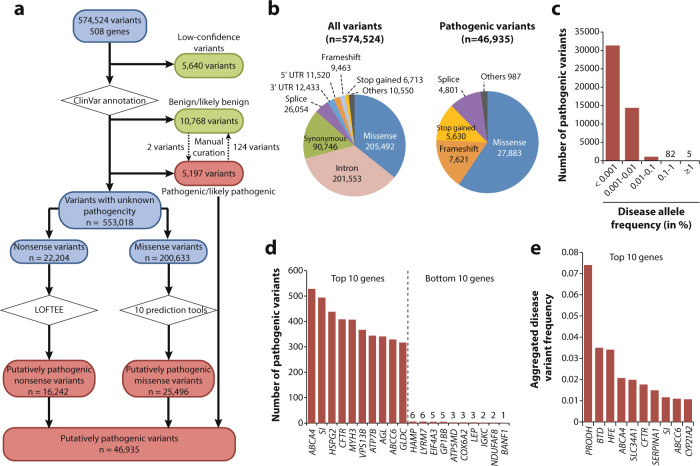
The landscape of pathogenic variability differs drastically between genes. **a** Algorithm for pathogenic variant selection in human autosomal recessive disease genes. In total, 574,524 variants were identified in 508 genes associated with autosomal recessive disease. In a first step, we removed low-confidence and benign variants, whereas all variants in these genes that were known to be pathogenic according to ClinVar (*n* = 5197) were included for further analyses. Rare variants with unclear pathogenicity were analyzed computationally using LOFTEE for nonsense variants or ten computational prediction tools for missense variants (see “Methods” section for details). Variants that were deemed as pathogenic by LOFTEE (*n* = 16,242) or all missense prediction algorithms (*n* = 25,496) were combined with the variants with pathogenic ClinVar annotation to yield the final data set of 46,935 pathogenic variants. **b** Pie charts visualizing the distribution of variant classes among all variants (left) and only among pathogenic variants identified in autosomal recessive disease genes (right). **c** The vast majority of pathogenic variants were very rare with global frequencies <0.001%, whereas only 82 and 5 pathogenic variants had frequencies between 0.1–1% and ≥1%, respectively. **d** Column plot showing the ten autosomal recessive disease associated genes with the highest and lowest number of pathogenic variants. Note that inter-gene differences in variant numbers exceed 500-fold. **e** The ten genes with the highest aggregated frequency of pathogenic variants are shown.

Across 508 genes associated with 450 AR diseases, we identified a total of 574,524 variants of which 46,935 were putatively pathogenic (Fig. 1a, b and Supplementary Data 1). The majority of all variants in these disease-associated genes were missense (*n* = 205,492; 35.8%), followed by intronic variations (*n* = 201,553; 35.1%), synonymous (*n* = 90,746; 15.8%), and splice site variants (*n* = 26,054; 4.5%). Of these, missense (*n* = 27,883; 59.4%), frameshift (*n* = 7,621; 16.2%), and stop gain variations (*n* = 5630; 12%) were strongly overrepresented among pathogenic variations, whereas intronic and synonymous variations were rarely associated with disease. As expected, the vast majority of pathogenic variations were very rare in the general population with minor allele frequencies (MAFs) < 0.001% (*n* = 31,358; 66.8%) and 28,185 (57%) were singletons (Fig. 1c). By contrast, only five disease-associated variations were found with global MAFs > 1% (rs2904552 in *PRODH*; rs1800562 in *HFE*; rs13078881 in *BTD*; rs1460573878 in *SLC34A1*; and rs28929474 in *SERPINA1*). The largest number of pathogenic variants were found in *ABCA4* (Stargardt disease; OMIM 248200; *n* = 528), *SI* (sucrase-isomaltase deficiency; OMIM 222900; *n* = 494), *HSPG2* (Schwartz–Jampel syndrome; OMIM 255800; *n* = 438) and *CFTR* (cystic fibrosis; *n* = 408), while 18 genes harbored less than ten pathogenic variants (Fig. 1d and Supplementary Data 2). When aggregating variant frequencies by gene, we find the highest frequency of pathogenic variants in *PRODH* (7.4%), associated with hyperprolinemia (OMIM 239500), followed by *BTD* (3.5%) and *HFE* (3.4%), which are linked to biotinidase deficiency (OMIM 253260) and type I hemochromatosis, respectively (Fig. 1e).

### Major differences in the global distribution of clinically important pathogenic variants

We profiled the variability in carrier frequency of pathogenic variants with the largest differences across ethnogeographic groups (Supplementary Data 3). For cystic fibrosis, p.Phe508del was the most common pathogenic variant with 248 and 112 per 10,000 individuals carrying at least one risk variant in Europeans and Ashkenazim, compared to 39.9 and 0 in South and East Asians, respectively. Similarly, the other common cystic fibrosis risk variants p.Gly542Ter, p.Gly551Asp, and p.Trp1282Ter were absent in Asian populations. Similar population differences were observed for the hemochromatosis variant rs1800562 (p.Cys282Tyr) in *HFE* for which carrier rates differed >300-fold across populations (1153 individuals per 10,000 in Europeans compared to 3 per 10,000 in East Asians). Additional examples of clinically important variations that were overrepresented >3-fold in Europeans include rs28929474 (p.Glu366Lys) in *SERPINA1*, rs116987552 (p.Arg50Ter) in *PYGM*, and rs1800546 (p.Ala150Pro) in *ALDOB*, which cause Alpha1 anti-trypsin deficiency (OMIM 613490), McArdle disease (OMIM 232600), and hereditary fructose intolerance (OMIM 229600), respectively.

By contrast, carrier frequency of rs334 (p.Glu6Val) in *HBB*, the most common causative variant of sickle cell anemia, was >30-fold higher in Africans compared to other populations. Our data furthermore corroborates the increased incidence of Gaucher disease (OMIM 230800), Tay–Sachs disease, and Canavan disease (OMIM 271900) in Ashkenazi Jews^10^. Notably, the molecular genetic underpinnings of Wilson disease (OMIM 277900), a disorder of copper metabolism caused by deficiency of *ATP7B*, also differed considerably between populations. While Wilson disease was attributed to rs76151636 (p.His1069Gln) in Ashkenazim (106 combined heterozygotes or homozygotes per 10,000 individuals) and Europeans (30.5 per 10,000 individuals), the population-specific variant rs28942074 (p.Arg778Leu) was underlying the disease in East Asians (37.9 per 10,000 individuals), corroborating the importance of considering ethnogeographic factors for disease allele profiling.

### Autosomal recessive disease prevalence can be accurately predicted based on population-scale sequencing data

**Fig. 2:**
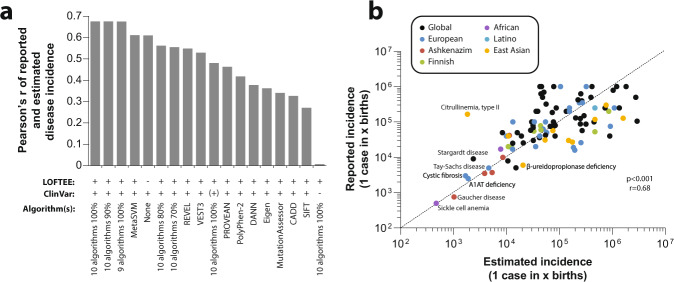
Disease prevalence predicted on population-scale genomic data closely corresponds to clinically reported disease frequencies. **a** Comparison of predictive performance of different prediction methods for 85 AR diseases. Note that integration of database information with stringent missense and nonsense predictions was overall most accurate, resulting in highly correlated predicted and reported disease incidence (Pearson’s *r* = 0.68; *p* < 0.0001). Note that when ClinVar annotations are not considered and disease incidence is purely calculated on the basis of computational pathogenicity assessments, the performance of the respective prediction model is very poor (*r* < 0.01). When only those pathogenic ClinVar variants are considered that are also estimated to be pathogenic using ten algorithms (indicated by “ClinVar (+)”), performance is strongly reduced (*r* = 0.48). **b** Detailed correlation of estimated and reported population-specific or global disease prevalence using the best performing prediction model from a ClinVar + ten algorithms + LOFTEE.

Next, we evaluated the prediction performance of database annotations and of the employed computational algorithms. To this end, we used a set of 85 AR diseases for which global or population-specific disease prevalence information was available and compared these values to the calculated incidences (Fig. 2a). Importantly, we find that the combination of curated ClinVar annotations with stringent predictions using ten algorithms predicted disease prevalences with the overall highest accuracy (Pearson’s *r* = 0.68, *p* < 0.0001; Fig. 2b). For instance, sickle cell anemia has been reported to occur in 1 in 500 individuals in African populations, which aligns well with predictions of 1 in 475. Similarly, frequencies of Gaucher disease (1 in 1028), Tay–Sachs disease (1 in 3884), and Canavan disease (1 in 8587), which are overrepresented in Ashkenazim, closely capture the reported frequencies of 1 in 750, 1 in 3500, and 1 in 9950, respectively. In addition, estimates of the integrated model for Wilson disease aligned well with its reported global prevalence based on clinical diagnostics (reported 1/7875; estimated 1/10,684). In contrast, associations based solely on curated ClinVar annotations or individual algorithms performed considerably worse (Pearson’s *r* = 0.27–0.61; Supplementary Data 4). To test whether the frequencies of certain variants might be inflated due to cohort selection, we moreover compared the frequencies of the ten most and least frequent diseases in the complete GnomAD data set to frequencies of subgroups, in which individuals with neurological phenotypes or cancer were excluded. Notably, differences in disease frequency were only very minor with correlation coefficients (Pearson’s *r*) >0.999 (Supplementary Data 5). Combined, these results indicate that computational modeling of AR disease prevalence that integrates variants with known pathogenicity with highly stringent functionality predictions, using methodologically diverse orthogonal in silico methods for rare and novel variants with unknown pathogenicity provides a powerful tool for the systematic analysis of population-specific AR disease risk.

### The global portfolio of pathogenic variation reveals striking differences in population-specific disease risk

Following the validation of our model, we then systematically mapped the prevalence of 450 AR disorders across seven major ethnogeographic groups, assuming a range of disease penetrance from 50–100% (Supplementary Data 6). Overall, the most prevalent AR condition was hyperprolinemia (OMIM 239500) with an estimated 54.6 cases per 10,000 individuals assuming 100% penetrance (Table 1). Hyperprolinemia was most prevalent in Ashkenazi Jews and Europeans, whereas the number of homozygous individuals was 660-fold lower in East Asians. The high frequency is primarily due to rs2904552 (p.Arg431His) in *PRODH*, which results in moderately (30–70%) reduced enzyme activity in vitro^11^. Notably, hyperprolinemia is relatively benign with the majority of homozygous individuals being asymptomatic^12^, suggesting that lower penetrance estimates for this condition are more appropriate. Similarly, in agreement with previous reports^13^, type I hemochromatosis, was >300-fold more common in Europeans and Finns compared to East and South Asians, primarily due to differences in the MAF of rs1800562 (p.Cys282Tyr) in *HFE*, as illustrated above. In contrast, pathogenic *ABCA4* variants underlying Stargardt disease was most prevalent in individuals of African ancestry, primarily due to the population-specific variant rs62642564 (p.Arg2107His)^14^. Multiple diseases were strongly overrepresented in a single population, including sickle cell anemia in Africans, as well as familial Mediterranean fever (OMIM 249100) and Gaucher disease in Ashkenazi Jews.

**Table 1:** Population-specific prevalence of ten common autosomal recessive disorders.

Disorder	Causative genes	Pathogenic variants^a^	Interethnic variability	Cases per 10,000 individuals							
				Global	EUR	FIN	AJ	LAT	SAS	EAS	AFR
Hyperprolinemia	PRODH, ALDH4A1	207 (7/200)	663	54.9	114.2	23.9	**132.5**	24.1	23.1	0.2	12.7
Hemochromatosis, type 1	HFE	35 (5/30)	335	11.6	**33.5**	12.3	1.4	2.4	0.1	<0.1	1.2
Biotinidase deficiency	BTD	124 (59/65)	309	12.2	18.4	**30.9**	10.8	5	17.7	<0.1	0.9
Stargardt disease	ABCA4	528 (149/379)	113	4.3	4.8	0.2	6.3	3	1.3	2.9	**22.5**
Sickle cell anemia	HBB	62 (44/18)	211	0.6	<0.1	0.1	<0.1	0.1	1.8	0.2	**21.1**
Familial Mediterranean fever	MEFV	95 (15/80)	155	0.1	0.1	<0.1	**15.5**	<0.1	<0.1	0.2	<0.1
Gaucher disease	GBA	93 (34/59)	98	0.1	0.1	0.1	**9.8**	<0.1	<0.1	<0.1	<0.1
Adrenal hyperplasia, congenital, due to 21-hydroxylase deficiency	CYP21A2	49 (10/39)	48	1.1	1.8	0.3	0.2	0.5	0.2	**9.6**	0.3
Infantile hypercalcemia	CYP24A1,SLC34A1	278 (19/259)	18	4.2	8.3	**8.8**	1.1	0.5	2.3	0.8	0.6
Cystic fibrosis	CFTR	408 (183/225)	39	3.1	6	0.6	**7.7**	2.2	1.2	0.2	1.3

Interethnic variability is defined as the fold change between the highest and the lowest population-specific frequency. The highest population-specific prevalence for each disease is indicated in bold.

*EUR* Europeans, *FIN* Finns, *AJ* Ashkenazi Jews, *LAT* Latin Americans, SAS South Asians, *EAS* East Asians, *AFR* Africans.

^a^Values in brackets indicate the number of pathogenic and number of predicted pathogenic variants.

The variability of pathogenic variation across populations was found to be surprisingly extensive. Of the 450 AR diseases analyzed, 101 (22.4%) were limited to specific populations and 172 (38.2%) and 325 (72.2%) diseases differed >100-fold and 10-fold in disease variant frequency, respectively, across the major ethnogeographic groups studied (Supplementary Data 6 and Supplementary Data 7).

### Genetic complexity of human autosomal recessive disorders

**Fig. 3:**
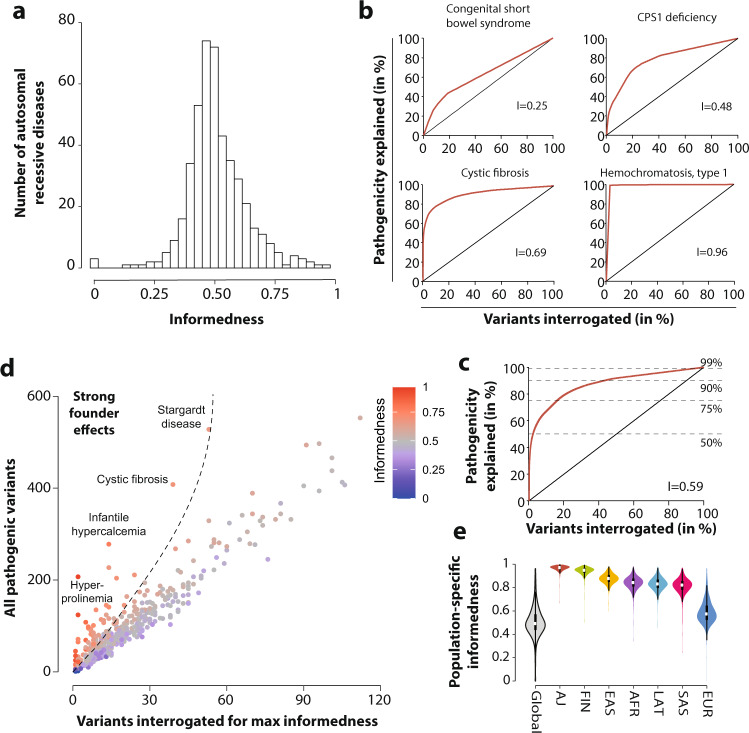
Genetic complexity and founder effects differ drastically across autosomal recessive diseases and populations. **a** Histogram showing the distribution of gene-specific informedness for 450 AR diseases. **b** Examples of receiver operating characteristic (ROC) curves of autosomal recessive diseases with high (type I hemochromatosis), medium (cystic fibrosis and CPS1 deficiency), and low informedness (congenital short bowel syndrome). **c** Aggregated ROC curve for all 450 analyzed diseases. Informedness values for all 450 analyzed diseases and the number of variants to be interrogated to capture 50, 75, 90, and 99% of disease cases are provided in Supplementary Data 6. **d** Color-coded scatter plot showing the association between the number of pathogenic variants per gene (ordinate) and the number of variants that need to be interrogated for maximal effectiveness of genetic testing (abscissa). The informedness for each disease is color-coded from blue (low informedness) to red (high informedness). Note that for certain diseases, such as hyperprolinemia, infantile hypercalcemia, and cystic fibrosis variants are strongly overrepresented, suggesting strong founder effects, whereas other diseases, such as persistent Mullerian duct syndrome and Brody myopathy show no indication of founder effects. **e** Violin plot showing population differences in informedness distributions. White dot indicates the median value of the distribution, while the black boxes indicate the spans between the first and third quartile.

We then analyzed the genetic complexity of all 450 AR disorders by computing the informedness of genetic testing. Specifically, we calculated the maximal excess of information that can be obtained by profiling a specific selection of candidate variants, in order to provide guidance for the optimal choice of genetic screening strategy. Overall, we find that informedness values cover almost the entire range of possible values from 0 to 0.96 (median = 0.49), with a leptokurtic (kurtosis = 2.8) positively skewed distribution (skewness = 0.19; Fig. 3a).

Examples of diseases with low informedness include congenital short bowel syndrome (OMIM 615237), which was associated with 27 pathogenic variants each of which had very similar explanatory power (*I* = 0.25; Fig. 3b). By contrast, stronger enrichment of disease risk across pathogenic variants was observed for carbamoylphosphate synthase I (CPS1) deficiency (OMIM 237300) and cystic fibrosis for which 23% and 10% of all disease-associated variants accounted for 70% and 78% of disease, respectively. The most genetically uniform disease was type I hemochromatosis for which 99% of cases was explained by a single variant (rs1800562 in *HFE*; p.Cys282Tyr; *I* = 0.96). Notably, p.His63Asp, a variant associated with altered iron status, does not substantially increase ferritin levels or cause clinically relevant organ iron overload^15^ and was not detected as pathogenic in our study. Aggregated across all analyzed diseases, we found that the profiling of 2.2% of pathogenic variants (*n* = 1015) was sufficient to detect 50% of disease-associated variability, whereas disproportionally higher numbers of variants would need to be interrogated for more complete coverage (Fig. 3c). The number of variants that need to be interrogated to detect 50, 75, 90, and 99% of disease cases for all 450 diseases analyzed is provided in Supplementary Data 8.

We then analyzed the landscape of AR diseases based on the total number of pathogenic variants, and the number of variants that needed to be interrogated to obtain the maximal excess in information for genetic screening (Fig. 3d). Diseases with a strong overrepresentation of one or few pathogenic variants can be indicative of founder effects^16^. Notably, we find strong evidence for such events in 29 diseases and syndromes, including hyperprolinemia, cystic fibrosis, and Stargardt disease, whereas other diseases, including persistent Mullerian duct syndrome (OMIM 261550) and Brody myopathy (OMIM 601003), were found to have very limited evidence of founder effects.

Lastly, we analyzed the population specificity of the informedness characteristic. We find that for most ethnogeographic groups the population-specific informedness was considerably higher compared to its global counterpart, corroborating results of substantial differences in the population-specific genetic constitution of the analyzed diseases (Fig. 3e). This was also true when only non-population-specific diseases were analyzed (Supplementary Fig. 1). Interestingly, informedness was overall higher in genetically more homogeneous populations (Ashkenazi Jews and Finns), demonstrating the reduced genetic complexity of AR diseases due to the genetic isolation. By contrast, informedness was lowest in Europeans, likely due to their relative overrepresentation in the utilized genomic databases.

## Discussion

Carrier screening for recessive disorders constitutes a well-established public health measure that has substantially decreased the burden of a variety of diseases, particularly in individuals belonging to certain genetic risk groups or with family history of genetic disease. Notable examples of the success of such screening programs when combined with pretest and posttest genetic counseling include the reduction of β-thalassemia (OMIM 613985) cases in Greece and Italy by 80–90% (ref. ^17^), and the diminishing of Tay–Sachs disease by ~90% in Ashkenazi Jews in the US and Israel^18^. Such population-scale genetic testing strategies require that the molecular genetics of the disease are understood and that carrier frequencies in the targeted population are well approximated.

Population-scale genetic screening relies on the interrogation of candidate variants, whereas sequencing is generally restricted to diagnostic applications. For instance, carrier screening for cystic fibrosis involves the testing of 23 *CFTR* variants, according to recommendations by the American College of Obstetricians and Gynecologists (ACOG) and the American College of Medical Geneticists (ACMG). However, this test panel can only explain ~80% of cystic fibrosis cases^19^, suggesting that rare non-tested genetic variation can have considerable impact on disease risk. Furthermore, very limited information on disease epidemiology and population specificity is available for most Mendelian disorders. Combined, this lack of data hampers studies of AR disease genetics and impedes the implementation of genetic screening programs for the majority of AR diseases. By integrating population-scale sequencing data of >140,000 individuals with a curated disease genetics knowledgebase and stringent computational analyses, we provide a comprehensive map of the human genetic AR disease landscape that considers both established pathogenic variants, as well as rare and novel variants with unknown pathogenicity. Based on the identified 46,935 pathogenic variants, we calculated disease prevalence and confidence intervals for 450 AR diseases, substantially extending previous prevalence estimates based on genomic data that only considered few selected diseases or gene families^20–23^.

Notably, the quality of population-specific disease prevalence based on genomic data critically relies on the accurate inclusion and exclusion of pathogenic and benign variants, respectively^24^. In an attempt to tackle these challenges, we calibrated our estimation model using epidemiological data of 85 AR diseases, and find that the most comprehensive ensemble method that integrates expert curated database information with stringent predictions for variants with unknown significance most accurately predicted clinical disease incidence (*r* = 0.68; *p* < 0.001). Furthermore, the translation of carrier frequencies into disease prevalence is complicated by variants with low clinical penetrance.

Current algorithms only flag variants as putatively pathogenic or benign, and can therefore not provide accurate estimations of the penetrance of a given variant. We thus provide disease frequency estimates for a range of overall penetrance values across diseases. While assuming penetrance of 100% is common in the incidence estimation of rare diseases^25^, the penetrance of a multitude of variations is considerably lower. For instance, type II citrullinemia, primarily allotted to the start-lost variant rs541276426 in *SLC25A13*, had clinical incidences that were 90-fold lower than predicted by our model. In vitro, the resulting truncated protein lacks transporter activity^26^ and has been described in patients with type II citrullinemia^27,28^; however, these findings are consistent with a low variant penetrance. Similar arguments apply to rs1800562 (p.Cys282Tyr) in in HFE as the causative variant for hereditary hemochromatosis^29^. As such, any predictions of clinically manifest disease prevalence can only serve as an approximation given a set of assumptions about variant-specific penetrance and that affected individuals are born and not lost in utero. Nevertheless, we believe that the data presented can provide novel insights about the genetic landscape of disease-associated genes and guide future follow-up work.

The aggregated frequency of pathogenic variations and genetic complexity of AR diseases was found to differ drastically across populations. Importantly, by using the excess of information that can be obtained from genetic testing of candidates (informedness), we provide comprehensive guidance for the choice of genetic markers across human AR diseases. Notably, we focus exclusively on genetic variation in exons or their very close proximity. As such, variants in noncoding regions that can be relevant for genetic disease, such as variants in the enhancers of *BCL11A* and *PTF1A* that cause Dias-Logan syndrome (OMIM 617101) and pancreatic agenesis (OMIM 609069), respectively, were not covered in our analysis^30,31^. Furthermore, our prevalence estimates only consider homozygotes and compound heterozygotes on a per-gene level, whereas the pathogenicity of polygenic variant combinations was not included.

While our study provides insights into population-variability and specificity of AR disease genetics in seven different major ethnogeographic groups, increases in geographic resolution promise to further refine analyses of the genetic disease structures. This is particularly true for isolated populations, in which founder mutations can cause high population-specific prevalence of genetic conditions and diseases, as shown for Inuit^32^, Druze communities in Israel^33^, as well as Dutch^34^ and Finnish populations^35^. These founder mutations are often not covered in conventional testing panels and, as a consequence, members of these isolated populations might not optimally benefit from pan-ethnic risk variant profiling^36^. Furthermore, resources, such as FINDbase, that aggregate and document the prevalence of clinically relevant genomic variation for populations and ethnic groups rather than along racial criteria provide important initiatives to increase the data reliability^8^.

In conclusion, the analyses presented here provide the most comprehensive analysis of the human AR diseasome and reveal striking differences in prevalence, genetic disease structure, and founder effects between major ethnogeographic groups. Our results pinpoint specific variant sets in each population that need to be interrogated for maximum potential effectiveness of genetic testing, thereby guiding the design of test panels and opening new avenues for precision public health.

## Methods

### Data sources

AR diseases and their corresponding causative gene associations were collected from the OMIM database. Specifically, we selected all AR diseases (i) for which the associated genes were not also associated with autosomal dominant traits, (ii) which were only associated with mutations in three or less different genes, and (iii) which are primarily caused by structural or noncoding genetic variation. Variants in disease associated genes and their population-specific frequencies were extracted from whole-genome and whole-exome sequencing (WGS and WES, respectively) data from 141,456 individuals worldwide (including 12,487 Africans, 17,720 Latinos, 5185 Ashkenazi Jews, 9977 East Asians, 15,308 South Asians, 12,562 Finns, 64,603 non-Finnish Europeans, and 3614 with unclear or other ethnicity), collated by the Genome Aggregation Database (gnomAD)^37^. Individuals included in the data were free from severe congenital disease. Data included in gnomAD were collected from multiple sources, where consent for aggregate release was obtained from donors. Because this data is made freely available to the public for aggregate, deidentified analysis, IRB approval specific to this study was not required.

### Identification of pathogenic variants

In total, 574,524 variants were identified in 508 genes associated with AR disorders, of which 5640 were removed due to low calling confidence (Fig. 1a). The remaining 568,884 variants were annotated for pathogenicity using ClinVar version 20190305. A total of 10,768 variants annotated as “benign” or “likely benign” were excluded, while variants annotated as “pathogenic” or “likely pathogenic” were considered as causative for disease. To anticipate the inflation of genetic risk estimation^38^, ClinVar variant classifications were manually curated using information from ACOG, ACMG, as well as the published literature, resulting in the reclassification of 126 variants and a total of 5197 pathogenic variants from database initiatives. The remaining 553,018 with unknown pathogenicity were computationally analyzed to predict their functional impacts. Variants leading to frameshifts, the loss of the start codon, premature stop codons, or that affected canonical splice sites were analyzed using LOFTEE (https://github.com/konradjk/loftee). Only variants that were classified as high-confidence loss-of-function variants by LOFTEE (16,242 out of 19,132; 85%) were considered as pathogenic. For the functional interpretation of missense variations, we used ten partly orthogonal computational prediction tools that performed best in different benchmarking datasets^39^, namely, SIFT, PolyPhen-2, MutationAssessor, VEST3, Eigen, CADD, DANN, MetaSVM, REVEL, and PROVEAN. Unless otherwise stated, only those variants were considered as pathogenic that were unanimously classified as such by all ten algorithms (25,496 out of 200,633; 12.7%). Combined, following the workflow illustrated above, we identified a total of 46,935 putatively pathogenic variations distributed across the 508 AR disease genes.

### Carrier frequencies and disease incidence estimations

Carrier frequencies and disease incidence was calculated based on the frequencies of all 46,935 putatively pathogenic variants. Disease incidence was calculated based on the Hardy–Weinberg equilibrium equation $p^2 + 2pq + q^2 = 1$, with *p* denoting the aggregated frequency of non-disease-causing alleles and *q* as the aggregated frequency of pathogenic alleles, thus considering both individuals homozygous and compound heterozygous for pathogenic variants. Disease incidence was calculated as $q^2 = [1 - \mathop {\prod }\nolimits_{i = 1}^n \left( {1 - f_i} \right)]^2$ based on the independence probability theory^40^, where *f* denotes the MAF of the pathogenic variant *i* in the respective gene. Based on the estimated genetic incidence, the prevalence of the disorders was estimated for penetrance levels of 100, 90, 70, and 50% for each disease.

### Estimation of genetic complexity

Informedness (*I*) was calculated using the Youden index to evaluate genetic AR disease complexity. Formally, informedness is calculated as *I* = max_*v*_ (*D*(*v*) − *P*(*v*)) based on the receiver operating characteristic curve of the fraction of interrogated pathogenic variants (*P*) for a disease plotted against the fraction of disease (*D*) that can be explained by this number of variants *v*, with *v* referring to a list *M* of all pathogenic variants in the respective gene sorted by MAF. The *v* value for which *I* is maximal (v*) denotes the optimal cutoff value for *M*, thereby providing the panel of variants with maximal effectiveness as a biomarker for the disease in question. An informedness value of *I* = 0 corresponds to an equal distribution of risk across disease-associated variants, while a value of *I* = 1 indicates that the entire disease risk can be predicted by profiling of a single variant.

### Reporting summary

Further information on research design is available in the Nature Research Reporting Summary linked to this article.

## Supplementary information

Supplementary Information

Supplementary Data 1

Supplementary Data 2

Supplementary Data 3

Supplementary Data 4

Supplementary Data 5

Supplementary Data 6

Supplementary Data 7

Supplementary Data 8

Reporting Summary

## Data Availability

All putatively pathogenic variants analyzed in this study are provided in Supplementary Data 1. Variant data of all 141,456 individuals is available at https://gnomad.broadinstitute.org/.

## Code availability

No custom code or scripts were used for the generation or processing of datasets.
