# Supplementary material for: The prevalence, genetic complexity and population-specific founder effects of human autosomal recessive disorders
Source: NPJ Genom Med. 2021 Jun 2;6:41. doi: 10.1038/s41525-021-00203-x (PMC8172936; doi:10.1038/s41525-021-00203-x)
Supplement: Supplementary file 1 — Supplementary Information [file 41525_2021_203_MOESM1_ESM.pdf]

The prevalence, genetic complexity and population-specific founder effects of human autosomal recessive disorders

Qingyang Xiao and Volker M. Lauschke

Supplementary Material

Supplementary Data: 8

Supplementary Figures: 1

Supplementary Data 1: Overview of all pathogenic variants analyzed. Genomic location, variant type, population-specific minor allele frequencies (MAF) and inclusion criteria are provided for all 46,935 pathogenic variants.

Supplementary Data 2: Overview of the number of pathogenic variants per analyzed gene.

Supplementary Data 3: Population-specific frequencies of the 30 disease-causing variants with largest ethnogeographic variability. Variability was calculated as the highest divided by the lowest number of variant carriers. For variants where the lowest number of variant carriers was zero, variability was set to 100. EUR = Europeans; FIN = Finns; AJ = Ashkenazi Jews; LAT = Latin Americans; SAS = South Asians; EAS = East Asians; AFR = Africans.

Supplementary Data 4: Clinically reported disease frequencies and predicted prevalence based on population-scale genomic data. These data form the basis for the correlation shown in Figure 2.

Supplementary Data 5: Comparison of carrier frequencies in the total gnomAD cohort compared to non-cancer and non-neuro subgroups. The top 10 most common and least common diseases are shown.

Supplementary Data 6: Estimated population-specific disease prevalence for 450 autosomal recessive diseases. EUR = European; FIN = Finnish; SAS = South Asian; EAS = East Asian; LAT = Latino; AFR = African; AJ = Ashkenazi Jews.

Supplementary Data 7: List of diseases that were limited to specific populations. The list includes all diseases shown in Supplementary Table 6 that have a frequency of zero in at least one population.

Supplementary Data 8: Overview of the informedness and number of variants that need to be analyzed to explain given fractions of associated disease cases.

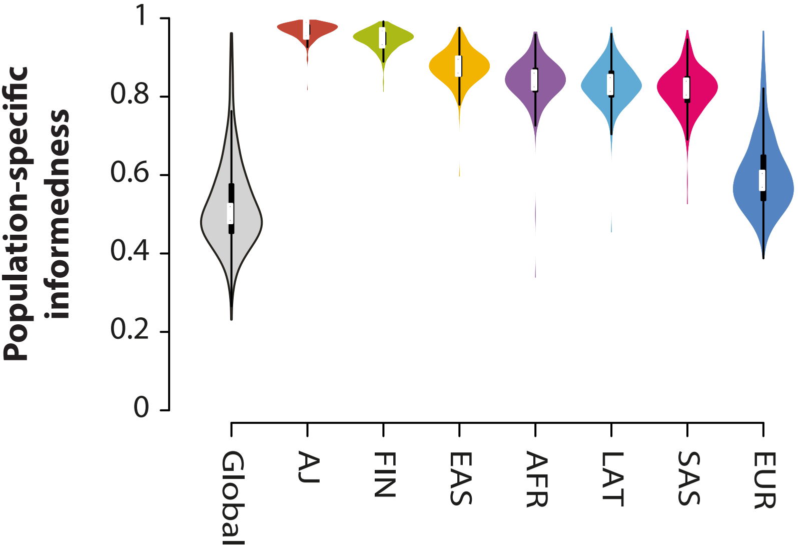

Supplementary Figure 1: Population informedness distributions are not driven by population-specific diseases. Violin plot showing population differences in informedness distributions when population-specific diseases are excluded. Note that exclusion of population-specific diseases results in only minor differences (compare Figure 3e). White dot indicates the median value of the distribution while the black boxes indicate the spans between the 1st and 3rd quartile.
